# Behavioral tests for the assessment of social hierarchy in mice

**DOI:** 10.3389/fnbeh.2025.1549666

**Published:** 2025-03-05

**Authors:** Hao Zheng, Dantong Chen, Zilong Zhong, Ziyi Li, Meng Yuan, Zhenkun Zhang, Xiaoping Zhou, Guohui Zhu, Hongwei Sun, Lin Sun

**Affiliations:** ^1^Department of Psychology, Shandong Second Medical University, Weifang, China; ^2^Clinical Medicine, Shandong Second Medical University, Weifang, China; ^3^Network Information Center, Shandong Second Medical University, Weifang, China; ^4^Depression Treatment Center, Weifang Mental Health Center, Weifang, China

**Keywords:** hierarchy, resource competition, social behavior, behavioral assessment, mental disorders

## Abstract

Social hierarchy refers to the set of social ranks in a group of animals where individuals can gain priority access to resources through repeated social interactions. Key mechanisms involved in this process include conflict, social negotiation, prior experience, and physical advantages. The establishment and maintenance of social hierarchies not only promote group stability and well-being but also shape individual social behaviors by fostering cooperation and reducing conflict. Existing research indicates that social hierarchy is closely associated with immune responses, neural regulation, metabolic processes, and endocrine functions. These physiological systems collectively modulate an individual’s sensitivity to stress and influence adaptive responses, thereby playing a critical role in the development of psychiatric disorders such as depression and anxiety. This review summarizes the primary behavioral methods used to assess social dominance in mice, evaluates their applicability and limitations, and discusses potential improvements. Additionally, it explores the underlying neural mechanisms associated with these methods to deepen our understanding of their biological basis. By critically assessing existing methodologies and proposing refinements, this study aims to provide a systematic reference framework and methodological guidance for future research, facilitating a more comprehensive exploration of the neural mechanisms underlying social behavior. The role of sex differences in social hierarchy formation remains underexplored. Most studies focus predominantly on males, while the distinct social strategies and physiological mechanisms of females are currently overlooked. Future studies should place greater emphasis on evaluating social hierarchy in female mice to achieve a more comprehensive understanding of sex-specific social behaviors and their impact on group structure and individual health. Advances in automated tracking technologies may help address this gap by improving behavioral assessments in female mice. Future research may also benefit from integrating physiological data (e.g., hormone levels) to gain deeper insights into the relationships between social status, stress regulation, and mental health. Additionally, developments in artificial intelligence and deep learning could enhance individual recognition and behavioral analysis, potentially reducing reliance on chemical markers or implanted devices.

## 1 Introduction

Social hierarchy is defined as a stable order established through repeated social interactions among individuals within a group, determining each individual’s rank or status, typically based on variations in body size or displays of aggression (Chase, 1974; Rowell, 1974). This hierarchical structure has been conserved throughout evolution and is prevalent in both animal and human groups (Lahn, 2020). Dominance relationships are often maintained through aggressive behaviors, where dominant individuals exhibit assertive actions, while subordinate individuals show signs of submission and retreat. These signals are frequently communicated visually, which contributes to establish and reinforce social order, thereby reducing prolonged and potentially harmful or lethal conflicts among group members (Sapolsky, 2005). Consequently, social hierarchies provide adaptive advantages to the group by structuring access to critical resources—such as food, territory, and mating opportunities—particularly in environments where resources are limited (Šabanović et al., 2020). Research has shown that the constraints on ranking disparities and the internalization of cooperative norms within a hierarchy can enhance group stability and cooperation, fostering an optimal balance between competition and collaboration. This not only reduces conflicts but also improves the overall health and reproductive efficiency of the group (Lozano et al., 2020; Strauss and Holekamp, 2019). This system effectively minimizes conflicts among conspecifics living in close proximity, and its formation is relatively straightforward (Hangyu et al., 2023), having profound effects on the health and disease outcomes of both animals and humans.

Social hierarchies are observed in a wide number of species across the animal kingdom. The assessment of the social hierarchies varies across species due to differences in behavioral expressions and physiological mechanisms. For instance, in crustaceans, such as shrimp and crabs, social rank is primarily determined by body size, claw dimensions, aggressive behaviors, and chemical signaling. Body size and claw dimensions generally dictate an individual’s dominance status, while aggressive displays (e.g., claw spreading) and pheromone release help reduce in-group conflict (Hangyu et al., 2023). In fish, biological complexity increases and social hierarchies are distinguished by a set of specific behavioral indicators including chasing, biting, fleeing (Arnott and Elwood, 2009), lateral threat displays (Desjardins et al., 2012; Hirschenhauser et al., 2004), and eye-bar activation (Oliveira et al., 1998). These behaviors serve as reliable predictors of male dominance (Chase et al., 2002; Oliveira et al., 1998). In birds, social rank is typically established through aggressive behaviors (e.g., pecking), threats, displacement, and chasing, often resulting in a linear hierarchy from dominant to subordinate individuals (Schjelderup-Ebbe, 1922). In primates, the assessment of social hierarchy is notably complex, involving the observation of aggressive and submissive behaviors (Walters, 1987), grooming interactions (Cheney, 1992; Seyfarth, 1977), group hugging, and spatial positioning within the group (Simonds, 1965). Additionally, behaviors such as drinking and feeding (Bernstein, 1963), infant-carrying (Zhang et al., 2006), and mounting (Furuya, 1957) serve as important indicators of social rank. These behaviors reflect marked asymmetries in social interactions, establishing core criteria for determining social hierarchy.

In laboratory research, the establishment and assessment of social hierarchies in mice provides an important experimental model for studying the impact of social rank on individual health, behavior, and physiological functions (Costa et al., 2021). Male mice, being inherently territorial, establish dominance hierarchies when forced to live together (Balog et al., 2019). Male mice establish social hierarchies through several behavioral mechanisms. The first strategy is physical aggression and fighting. Male mice typically determine dominance through direct physical confrontations, with stronger individuals often winning these encounters and thus attaining higher social status (Barnett, 2017). The second strategy involves threat displays. These behaviors include baring teeth, raising fur, and vocalizing, which allow male mice to establish dominance without engaging in actual combat (Blanchard and Blanchard, 1989). Additionally, scent marking (via urine, feces, or glandular secretions) and social interactions (such as grooming and nose touching) also play a crucial role in the establishment and maintenance of social hierarchies, with higher-ranking individuals receiving more frequent attention and grooming from group members (Calhoun, 1963). Finally, resource control is key to establishing social rank. Individuals that control essential resources like food, water, and nesting sites typically achieve higher social status. The ability to control and distribute resources is a vital component of social hierarchy (Wolff, 1993). Access to resources exhibits pronounced asymmetries in social groups, representing a fundamental criterion for the assessment of social hierarchies.

Recent research in humans increasingly acknowledges social status as a critical determinant of mental health disorders such as depression (Hardman et al., 2023; Vidal et al., 2023), anxiety (Kessler et al., 1999), and post-traumatic stress disorder (Brewin et al., 2000). Epidemiological evidence has consistently demonstrated a strong association between lower socioeconomic status (SES) and poorer physical and overall health outcomes (Adler et al., 1994; Cundiff and Matthews, 2017; Quon and McGrath, 2014; Singh-Manoux et al., 2005; Tan et al., 2020). However, other studies suggest that individuals from lower SES backgrounds may exhibit higher resilience to stress, challenging the traditional view that social disadvantage is solely a risk factor for poor mental health. Indeed, lower SES individuals often demonstrate adaptive coping mechanisms, such as enhanced emotional regulation and the use of strong social support networks, which may help to buffer the negative effects of stress (Buheji, 2020; Pastwa-Wojciechowska et al., 2021). Therefore, when understanding the impact of social status on mental health, it is essential to consider its multifaceted effects, rather than viewing it solely as a predictor of mental health problems.

Further research in mice has elucidated the role of social rank in modulating brain neuroactivity and emotional behaviors. Studies indicate that high-ranking mice typically exhibit reduced dopamine neuron activity, which correlates with stronger resilience, lower susceptibility to depressive-like behaviors, and enhanced cognitive functions. Conversely, mice of lower social status display increased anxiety levels and heightened sensitivity to addictive substances (Battivelli et al., 2024). Notably, reduced firing rates of pyramidal neurons and diminished *γ*-oscillation activity in the medial prefrontal cortex (mPFC) are observed in low-ranking mice, linking these neural changes to their depressive and anxious behaviors. Chronic stress further exacerbates emotional disorders and suppresses neuronal activity in the mPFC of these low-ranking individuals (Yin et al., 2023). Additionally, social status has been shown to influence anxiety levels through the modulation of gut microbiota and their metabolites, particularly under conditions of chronic pain, where subordinate mice exhibit more pronounced anxiety behaviors (Wang et al., 2024). Interestingly, while high-ranking mice generally exhibit greater resilience and superior cognitive abilities, they may be more susceptible to depression-like behaviors under certain conditions. Studies have shown that after experiencing chronic social defeat stress, dominant mice display heightened vulnerability, characterized by pronounced social avoidance behaviors, whereas subordinate mice remain largely unaffected. This suggests that the pressures of maintaining dominance or the psychological impact of status loss may pose unique challenges to dominant individuals (Larrieu et al., 2017; Larrieu and Sandi, 2018). However, research using the non-social nature of the chronic unpredictable stress model offers a different perspective, indicating that the vulnerability of high-ranking individuals may not be entirely attributed to the loss of social status but could involve other stress-related mechanisms (Cherix et al., 2020). These findings highlight the importance of exploring the complex relationship between social status and stress responses, providing new insights into the mental health risks associated with different social ranks.

Laboratory research using mice has become an indispensable method for exploring the neural mechanisms underlying social dominance behavior. To uncover the processes driving the formation of social hierarchies, a robust and reliable behavioral assessment is essential. For example, Fulenwider et al. (2022) summarized key methodologies for assessing social dominance in laboratory rodents, categorizing them into paradigms based on agonistic behaviors (e.g., the resident-intruder test) and those based on resource competition (e.g., food competition tests). Their analysis highlighted the broader applicability of resource-based assays across sexes and species, while emphasizing the notable gap in research involving female rodents. This gap underscores the need for more comprehensive investigations, particularly into how social hierarchies are established and maintained in different sexes. Building on these foundational insights, this review expands the discussion by analyzing commonly employed methods for assessing social dominance, introducing emerging approaches, and evaluating their strengths and limitations. Additionally, we delve into the neural mechanisms underlying these methods, aiming to provide a robust framework and guidance for future research in the study of social hierarchies.

## 2 Major classification methods

### 2.1 Tube test

In the study of social hierarchy in mice, the Tube test (Wang et al., 2014) is one of the foundational experimental methods. This test operates on principles akin to social interactions in narrow corridors: two mice enter from opposite ends of a tube, forcing them to meet in the middle and compete for passage. Lindzey et al. (1961) were among the first to effectively assess social ranking in inbred mouse strains using this experimental design. In their study, mice were food-deprived to maintain their body weight at 85% of normal levels. Following training, when the mice encountered each other in the tube, the winner could push the opponent out and gain access to food rewards.

**Figure 1:**
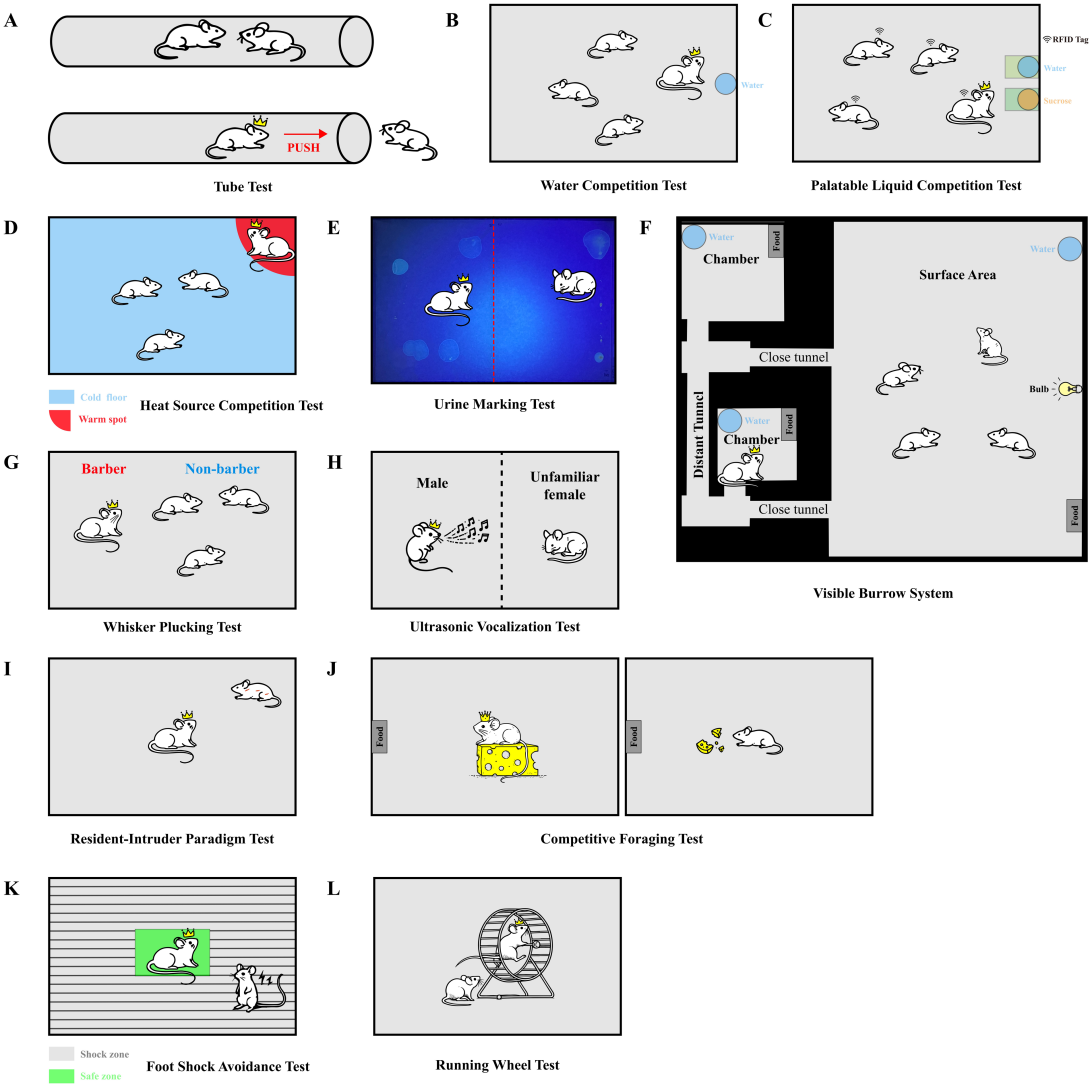
**(A)** Tube test: the tube test is used to assess social dominance in mice by forcing two individuals to compete for passage through a narrow tube. The winner is the mouse that successfully induces the opponent to retreat and to exit the tube from the side opposite to the opponent. **(B)** Water competition test: this test assesses social dominance by measuring the time mice spend drinking from a shared water source, with more dominant individuals having longer access. **(C)** Palatable liquid competition test: dominant males show greater access to a palatable liquid. **(D)** Heat source competition test: mice compete for access to a warm spot in a cold environment. The amount of time spent occupying the warm area reflects social rank, with higher-ranking mice occupying it longer. **(E)** Urine marking test: the urine marking test evaluates social dominance by analyzing the spatial distribution of urine markings within a shared environment, with dominant mice typically marking central areas. **(F)** Visible burrow system: the VBS simulates a natural burrow environment, allowing researchers to observe social interactions and rank formation within mouse groups under semi-natural conditions. **(G)** Whisker Plucking test: the whisker plucking test identifies dominant mice by observing the removal of whiskers from subordinate cage mates, reflecting their higher social rank. **(H)** Ultrasonic vocalization test: this test measures ultrasonic vocalizations (USVs) emitted by male mice during courtship of females, with higher frequency and complexity of calls correlating with increased social dominance. **(I)** Resident-intruder paradigm test: the resident-intruder test assesses social aggression by introducing an unfamiliar intruder into the home territory of a resident mouse, measuring the aggressive behaviors displayed by the resident. **(J)** Competitive foraging test: this test evaluates social rank based on the ability of mice to compete for food within a limited time, with dominant mice securing more food. **(K)** Foot shock avoidance test: mice compete for a single escape platform to avoid foot shocks, with the dominant mouse typically gaining control of the platform and avoiding the shocks. **(L)** Running wheel test: this test measures the frequency of running wheel use among mice, with more dominant individuals using the wheel more frequently, indicating a higher social status. **(A–L)** The crown symbol highlights the dominant animal.

Hu and colleagues (Fan et al., 2019) further simplified this experiment by employing a transparent plastic tube, eliminating the need for goal boxes and complex gate structures. Their research demonstrated that the presence or absence of food deprivation did not significantly impact the results, thereby allowing the removal of food deprivation from the protocol, leading to optimization of animal welfare and reduction of confounding variables (Wang et al., 2011) (Figure 1A). Additionally, a slit can be incorporated into the tube for real-time optogenetic manipulations, *in vivo* electrophysiological recordings, or calcium imaging during testing.

The standard dimensions of the tube test are approximately 30 cm in length and 3 cm in diameter, but can be adjusted according to the size of the experimental animals. During the experimental process, animals should undergo prior acclimatization in the tube to reduce stress and mitigate potential “winner effect” and “loser effect.” Through a series of repeated trials (Wang et al., 2011) and a scoring system (such as David’s Score, DS) (de Esch et al., 2015; Gammell et al., 2003; van den Berg et al., 2015), researchers can determine the social ranking of mice and evince their social status within the group. Additionally, automated tube test systems, such as the one provided by Benedictus Systems (Rotterdam, the Netherlands), can effectively reduce human interference.

The tube test offers significant advantages in measuring social hierarchy. It is simple to operate, requiring only a plastic tube, and effectively avoids intense conflicts, thereby reducing animal injury (Fan et al., 2019). The results are reliable, with nearly all mice obtaining a stable hierarchical ranking. However, the test also has limitations. Results may be influenced by confounding factors such as the mice’s age, weight, and baseline stress levels, necessitating careful matching and training (Larrieu et al., 2017; Park et al., 2018). It is important to note that this testing method itself can impose stress on the mice. Studies have shown that male and female mice, after repeatedly losing in the tube test, begin to exhibit passive coping behaviors (Fulenwider et al., 2024). Additionally, different rodent strains may exhibit behavioral variations, so it’s essential to validate the test’s correlation with other ranking methods before applying it to new species. Finally, the method of victory must be considered; for instance, rats treated with tetrahydrocannabinol (THC) may win by exhibiting “freezing” behavior, making it crucial to quantify actions like pushing, resisting, and retreating (Miczek and Barry, 1974).

Recent studies have further explored the neural mechanisms underlying social interactions in the tube test. Research highlights the critical role of the mPFC in regulating social dominance, a finding that has been confirmed in both mice (Zhou et al., 2017) and humans (Ligneul et al., 2016; Zink et al., 2008). Within the mPFC, distinct types of neurons in the dorsomedial prefrontal cortex (dmPFC) play different roles during social competition. Specifically, pyramidal (PYR) and vasoactive intestinal peptide (VIP) neurons are activated during pushing behaviors, while parvalbumin (PV) neurons are notably active during both pushing and retreating, indicating their dual function in competition. Optogenetic and chemogenetic manipulations reveal that inhibiting PV neurons enhances competitive behavior, whereas inhibiting VIP neurons reduces it (Zhang et al., 2022). Furthermore, neural activity in the prelimbic (PrL) region correlates closely with performance in competitive tasks, with dominant animals showing higher ΔF/F signals during pushing and subordinate animals showing lower signals during retreat. This dynamic shift in neural activity is closely tied to behavioral transitions (Garcia-Font et al., 2022). Additionally, the projection pathway from the ventromedial prefrontal cortex (vmPFC) to the nucleus accumbens shell (NAcSh) is modulated by the social rank of competitors. Studies have shown that this pathway exhibits heightened activation when mice face more dominant opponents, highlighting its critical role in regulating dominance behaviors. Furthermore, this pathway plays an essential role in behavioral recovery following social defeat, offering new insights into its dual function in the regulation of social hierarchy (Fetcho et al., 2023).

### 2.2 Resource-based competition tests: water and heat source competition

The Water Competition Test, originally developed by Bruce (1941), is a widely used method to assess social hierarchy in mice. In this test, a water bottle with a shielded drinking spout is placed in the experimental setup, allowing only one mouse to access the water at a time. In situations where two or more mice compete within a limited timeframe, the individual that controls the drinking spout for the longest period is considered the dominant one, thereby indicating a higher social rank (Figure 1B). Typically, the competition duration ranges from 2 to 15 min, with the entire drinking process being recorded via camera to measure each mouse’s drinking time. To enhance the discriminative power of this test, animals are often subjected to water deprivation beforehand, with deprivation periods ranging from 5 to 24 h (Syme et al., 1974).

A related approach is the Palatable Liquid Competition Test, which also serves as an indicator of social rank. Dominant individuals generally consume more palatable resources than their lower-ranking counterparts (Gentsch et al., 1990; Merlot et al., 2004). This test can be conducted in specialized cages (e.g., HM2) equipped with Radio-Frequency Identification (RFID) chips to accurately track liquid consumption in group-housed environments (Fulenwider et al., 2021; Johnson et al., 2024). High-attraction liquids, such as sugary solutions, are provided, and RFID chips record each mouse’s liquid intake and frequency, effectively reflecting its social standing (Figure 1C). Although this test is highly motivating and provides multidimensional data, it requires advanced equipment and incurs relatively high costs.

In contrast, the water competition test is simpler, primarily involving the use of a water bottle with a shielded spout and video recording to monitor the drinking behavior of mice. This method is easy to conduct, highly reproducible, and allows for a straightforward assessment of social hierarchy based on drinking time and frequency. However, the water competition test focuses mainly on drinking behavior, potentially overlooking other important social behaviors. Additionally, water deprivation may increase stress in mice, potentially affecting the experimental outcomes. The palatable liquid competition test is well-suited for studies requiring high motivation and multidimensional data, whereas the water competition test is more appropriate for simple, rapid assessments of social hierarchy.

Building on similar physiological motivations, the warm spot test (Zhou et al., 2017) also induces competition among mice by limiting access to a crucial resource. In this test, multiple mice are placed in a cold chamber (0°C) where only one corner is warm (34°C). The mice remain in the chamber for 20 min, during which their behaviors are recorded, with a focus on competition for the warm corner (Figure 1D). Due to their innate drive for warmth, mice view the warm corner as a valuable resource, and individuals that occupy this area for longer are inferred to have higher social status (Ganeshan and Chawla, 2017; Kumar et al., 2009). The warm spot test is highly motivating and captures natural behaviors, showing strong concordance with tube test results and further validating its reliability (Zhou et al., 2017). However, prolonged exposure to cold may increase stress, so adequate warming is needed after the test to ensure animal welfare.

### 2.3 Urine marking method

Mice communicate their individual identity and social status through major urinary proteins (MUPs), which confer a unique volatile odor signature. Studies have shown that females rely primarily on MUP profiles rather than MHC genotypes when learning and recognizing scents; notably, dominant (*α*) male mice express significantly higher levels of MUPs—particularly MUP20—than their subordinate counterparts (Roberts et al., 2018). These differences emerge within just 1 week of social hierarchy formation, suggesting that dominant individuals undergo physiological changes to enhance their odor-based signaling, potentially reflecting the energetic demands of maintaining their status (Lee et al., 2017).

Building on these physiological differences, urine marking—an essential means of communication and social interaction among mice—also represents a useful behavior to determine hierarchical patterns. In experimental setups, two previously isolated male mice are paired in a neutral, clean enclosure with filter paper at the base to visualize urine markings under ultraviolet light (Figure 1E). To accurately quantify urine marking, a transparent grid overlay can be placed over the filter paper, and the number of grid units containing urine marks can be counted, allowing for standardized measurement and comparison of urine marking behavior between individuals (Drickamer, 2001; Sipos and Nyby, 1996). Upon initial contact, these mice typically engage in an aggressive encounter. By separating the two mice with a divider, researchers collect urine over periods ranging from 2 to 22 h. The results indicate that dominant mice concentrate their urine markings in the center of the enclosure, while subordinate mice primarily mark the corners and edges (Desjardins et al., 1973; Horne and Ylönen, 1996; Horne and Ylönen, 1998). This dominant-subordinate marking pattern is less pronounced in group-housed mice, likely due to reduced aggression in stable social hierarchies (Wang et al., 2011). Nevertheless, among cage mates, higher-ranked mice in tube tests tend to urinate more frequently and closer to the divider (Wang et al., 2011). It is important to note that urine marking is primarily used to differentiate social hierarchies among male mice, with insufficient evidence that it plays a similar role in females. Moreover, in free-living root voles (*Microtus oeconomus*), the relationship between male social dominance and traits such as testosterone levels and urine marking remains inconclusive (Borowski et al., 2014). Consequently, caution should be exercised when using scent marking as a proxy for social rank, and it is advisable to supplement it with other behavioral assays to achieve a more accurate assessment of an individual’s social status.

Male mice process urine signals through key brain regions, including the medial amygdaloid nucleus (MEApv) and the main olfactory bulb (MOB), with MEApv responding strongly to dominant male urine (Roberts et al., 2018). Social rank and scent familiarity influence activity in the ventromedial hypothalamus (VMH), premammillary ventral nucleus (PMv), and ventrolateral periaqueductal gray (vlPAG), shaping adaptive social responses. On the other hand, the lateral hypothalamus (LHA) regulates urine marking via the pontine micturition center (PMC), ensuring appropriate territorial signaling (Hyun et al., 2021). Overall, these neural mechanisms allow male mice to communicate hierarchy in social environments.

### 2.4 Visible burrow system

The Visible Burrow System (VBS), developed by Robert and Caroline Blanchard at the University of Hawaii (Blanchard R. J. et al., 1985), is an experimental model designed to simulate a natural burrow environment. Widely used to study social behaviors and stress responses in rodents (Choi et al., 2006; Smeltzer et al., 2012), the VBS facilitates the formation of stable social hierarchies in mixed-sex rat groups (generally four male and two female adult rats), typically consisting of one dominant individual and several subordinates (Blanchard et al., 1995; Melhorn et al., 2017). The system consists of a large open surface area (SFC) connected to small, darkened chambers via tunnels, mimicking a semi-natural environment. Transparent plexiglass, combined with infrared lighting and video surveillance, allows researchers to monitor the rats’ activities in darkness, offering a unique way to observe natural behaviors over extended periods (Blanchard et al., 1995). This setup enables long-term monitoring of natural behaviors, allowing researchers to accurately distinguish social ranks within the group and examine how these hierarchies influence behavior, neurophysiology, and the endocrine system (Figure 1F).

In the VBS, male rats establish social hierarchies through complex social interactions, with researchers evaluating social status based on multiple behavioral and physiological indicators. Aggressive behaviors, such as chasing and biting, serve as primary markers, with higher-ranking individuals typically engaging in more frequent aggression to maintain dominance. Dominant individuals also have priority access to resources, including food, water, and resting areas, while subordinate individuals exhibit avoidance or submissive behaviors during competition. Social interactions and spatial usage further reflect social rank, with dominant rats occupying prime spaces and initiating more social contact, while lower-ranking rats show avoidance and isolation.

Importantly, sex plays a significant role in the formation of social hierarchies. While male rats form distinct social hierarchies, female rats do not exhibit clear rank differentiation in the VBS (Blanchard R. et al., 1985). Additionally, stress responses serve as key physiological indicators. Subordinate males often display chronic stress markers such as weight loss and elevated cortisol levels, as well as behaviors associated with anxiety and depression (Tamashiro et al., 2005). Studies have shown that dominance in the VBS correlates significantly with rankings in tube tests (Blanchard et al., 1995; Wang et al., 2011).

While aggression is typically considered the primary determinant of social rank, research indicates that aggression alone does not fully explain hierarchy formation. Some less aggressive individuals achieve dominance through alternative strategies. For example, in Wildtype Groningen rats, weight loss and bite wounds in subordinates do not correlate significantly with aggression levels (Buwalda et al., 2017). Unlike traditional stress models like restraint or foot shock, the VBS imposes stress through naturally occurring social interactions. Consequently, dominance hierarchies form organically without experimental manipulation. This makes the VBS a valuable tool for studying social stress in a context resembling natural environments (Tamashiro et al., 2004). Currently, few studies have employed the VBS as a method for distinguishing social hierarchies in mice, a limitation that may be attributed to the mice’s small body size and high mobility, which pose significant challenges for precise behavioral monitoring and data collection. To address these limitations, some studies have integrated video surveillance and RFID technology to automatically track multiple animals in semi-natural environments, thereby enabling accurate recordings of individual behavior, pairwise interactions, and group structures. In the future, incorporating these advanced techniques into VBS experiments is expected not only to enhance the accuracy and reliability of data collection but also to provide deeper insights into the intrinsic mechanisms underlying the formation of social hierarchies in mice, thereby offering new perspectives and methodologies for research in this field (Weissbrod et al., 2013).

### 2.5 Whisker plucking test

The whisker plucking test is a widely recognized method for assessing social hierarchies in mice. This method is valued for its simplicity, non-invasiveness, and its ability to clearly differentiate social roles. By observing whisker and fur trimming behavior among cage mates, researchers can identify dominant and subordinate individuals (Long, 1972; Militzer and Wecker, 1986; Sugimoto et al., 2018). In Long’s study (Long, 1972), it was observed that within groups of cohabiting mice, a specific individual often retains intact whiskers and fur, while others exhibit whisker loss or fur patches. When “barber” individuals from different cages are housed together, aggressive encounters frequently result in the victor plucking whiskers of the defeated individual. This suggests that whisker plucking typically occurs following conflict and is associated with dominance behavior. This phenomenon is known as the “Dalila effect,” where the dominant individual grooms and plucks the whiskers of its subordinates (Figure 1G) (Sarna et al., 2000). Interestingly, this is also commonly observed in female mice (Bresnahan et al., 1983; Kalueff et al., 2006). Moreover, research indicates a strong correlation between social ranking in the tube test and whisker plucking behavior (Hauschka, 1952; Kalueff et al., 2006; Strozik and Festing, 1981).

However, alternative interpretations challenge the exclusive link between whisker plucking and dominance. Some studies suggest that this behavior may also arise from environmental stressors (such as cage design, social dynamics, or sensory deprivation) rather than solely indicating dominance. For example, the presence of a “barber” in the cage can promote similar behaviors among cage mates, implying a role for social learning or reinforcement (Garner et al., 2004a). Notably, early research on rats associated the grooming drive with the desire to be groomed, arguing that this behavior was independent of social dominance. Given the limited reports using the Whisker Plucking Test to assess rat social hierarchies, caution is advised when using rats as experimental models. Moreover, some researchers question the validity of whisker plucking as a direct marker of social hierarchy, suggesting it may instead reflect aberrant repetitive behaviors akin to human trichotillomania (Latham and Mason, 2004). Additional studies indicate that whisker plucking might be linked to boredom or a lack of environmental enrichment, as improved housing conditions significantly reduce grooming and plucking behaviors (Bechard et al., 2011; Garner et al., 2004b; Kurien et al., 2005). In summary, while whisker plucking offers valuable insights into mouse social interactions, its complex etiology makes complementary methods necessary when evaluating social hierarchies.

### 2.6 Ultrasonic vocalization test for mating calls

Ultrasonic vocalizations (USVs) are an effective indicator for assessing social hierarchies in mice (Nyby et al., 1976). All vocalization characteristics, including amplitude and bandwidth (the frequency range spanned by the signal), can be recorded using ultrasonic-sensitive microphones and quantitatively analyzed with specialized software, for example, Avisoft Recorder (Scattoni et al., 2008). Each vocalization can also be qualitatively categorized according to its structure into one of 10 types: complex, harmonic, two-syllable, upward, downward, V-shaped, short, composite, frequency step, and flat calls (Scattoni et al., 2008). When dominant male mice encounter females, their courtship USVs are significantly higher in both frequency and occurrence compared to subordinate males (Figure 1H). Multiple studies have shown that the 70 kHz USV is a prominent feature in male courtship behavior and is closely associated with sexual motivation (Nyby et al., 1976; Whitney et al., 1974). Higher-ranking male mice emit more USVs and respond more rapidly to female stimuli, while lower-ranking males produce few, if any, USVs (Nyby et al., 1976; Wang et al., 2011).

Social environment plays a significant role in shaping USV patterns in mice. Mice living in enriched environments produce more diverse USVs, which reflect both their social interactions and hierarchical status (Peleh et al., 2019; Sangiamo et al., 2020). The neural and genetic foundations of USVs further support their role in reflecting social behavior and rank, as specific neural circuits and genetic factors influence the characteristics of these vocalizations (Yao et al., 2023). Notably, social and stress-related USVs are governed by distinct neural mechanisms. Social USVs, particularly those involved in mating and other social interactions, are primarily controlled by specific neuron populations in the periaqueductal gray (PAG) region of the midbrain. These neurons play a crucial role in regulating male mice’s courtship behavior and sexual motivation. Specifically, estrogen receptor 1-positive neurons in the lateral preoptic area (LPOA^ESR1^) dynamically regulate the amplitude, duration, and social context dependency of male mouse courtship USVs by disinhibiting USV-gating neurons in the PAG through a di-synaptic disinhibition pathway (Chen et al., 2021). In contrast, stress-related USVs, such as ultrasonic vocalizations triggered by pain or fear, are controlled by different neural circuits, potentially located in the brainstem or other areas, independent of the PAG neurons. Research shows that while disruption of PAG neurons affects social USVs, mice can still produce stress-related USVs, indicating that the neural circuits for social and stress-related USVs are independent of one another (Tschida et al., 2019; Ziobro et al., 2024).

Therefore, USV analysis is a powerful method for studying social structure and dynamics in mice. High-frequency and complex USVs are closely associated with dominant status, and this method is minimally invasive, causing relatively low stress to the animals. However, this approach has several limitations. Environmental noise and experimental conditions, such as temperature fluctuations and external disturbances, can significantly impact recording quality and data accuracy, underscoring the need for well-controlled experimental environments. To mitigate these challenges, recordings are often conducted in anechoic, sound-attenuating chambers (e.g., Med Associates Inc.) (Rieger and Dougherty, 2016), which effectively reduce background noise and external interference to ensure reliable data collection. Additionally, with advancements in technology, USV analysis tools such as DeepSqueak and VocalMat have been developed to enable efficient and automated detection and classification of USVs. In particular, DeepSqueak incorporates deep learning techniques to improve analytical accuracy and reduce background noise (Coffey et al., 2019), while VocalMat employs machine learning for high-precision classification without requiring complex user inputs (Fonseca et al., 2021). These tools provide researchers with practical methods to study mouse communication and behavior more effectively.

Furthermore, individual variability among mice, such as differences in vocalization propensity, can lead to incomplete data, as not all mice produce USVs during testing. For example, certain USV features, such as spectral characteristics like pitch jumps in adult mice, have been shown to exhibit low consistency across sessions, suggesting that these features are more susceptible to the animal’s state at the time of the recording (Rieger and Dougherty, 2016). This variability highlights the importance of combining USV analysis with complementary behavioral assays to obtain a more comprehensive understanding of social hierarchies and their underlying mechanisms.

As a complement, a newly developed Mate Competition Test, originally conducted using C57BL/6 mice, provides an alternative behavioral perspective for assessing social hierarchy in mice (Jing and Shan, 2023). This method evaluates male mice’s social status by observing their behavioral responses to female stimuli under controlled conditions. Similar to USV analysis, the Mate Competition Test focuses on social interactions and hierarchical behavior. While this test is still in the exploratory phase and requires further validation of its reliability and applicability, it offers a valuable behavioral complement by measuring the frequency and duration of male–female interactions.

### 2.7 Exploratory methods for studying social hierarchies in mice

Although the aforementioned methods hold a mainstream position in studying social hierarchies in mice, they also have certain limitations, particularly in capturing nuanced social behaviors. With the progression of research and the diversification of experimental requirements, some existing methods are being reevaluated and refined, while new methods are being developed to better meet specific experimental needs. For instance, the resident-intruder paradigm, while widely employed to investigate aggression and dominance behaviors in general contexts, remains relatively underutilized in studies specifically focusing on social hierarchies. By introducing an intruder into the territory of a resident mouse, this method triggers naturalistic social interactions, allowing researchers to quantify the aggressive behaviors of the resident (Figure 1I) (Koolhaas et al., 2013). Typically, dominant individuals exhibit higher levels of aggression; however, this is not an absolute rule, as the social behavior of mice demonstrates a certain degree of plasticity. In stable social group environments, aggression is often suppressed (Wang et al., 2014). It is worth noting that the outcomes in this paradigm are significantly influenced by factors such as body size, territoriality, and prior experience. If not controlled, these conditions may compromise the ecological validity of the model (Mooney et al., 2014).

The competitive foraging test assesses social status by measuring an individual’s ability to secure resources in a competitive setting, providing insights into hierarchical structures within groups based on resource control and competitive efficiency (Figure 1J) (Murtazina et al., 2023). The foot shock avoidance test, conducted in a confined apparatus with an escape platform, examines dominance-subordinate relationships in mice by observing which mice consistently claims the escape position under aversive conditions, thereby revealing rank-related behaviors in high-stress, competitive environments (Figure 1K) (Bevan et al., 1960). Finally, the running wheel test leverages spontaneous activity preferences to reflect social hierarchy in mice, as dominant mice are typically observed to have greater access to and usage of the running wheel, while subordinate mice exhibit avoidance behavior, likely due to social inhibition (Figure 1L) (Balog et al., 2019; Olsson and Sherwin, 2006).

These methods not only expand the scope of social behavior assessment in mice but also enable researchers to capture their subtle and multifaceted social dynamics from various perspectives. However, further validation is needed to ensure the applicability and reliability of these approaches across different strains of mice. In the future, integrating these emerging techniques with traditional methods is expected to provide a more comprehensive understanding of the complexity of social behavior in mice, thereby offering novel insights into the study of social hierarchies.

## 3 Discussion

The formation of social hierarchies is a naturally occurring and evolutionarily conserved phenomenon with profound implications for health and disease. Abnormal social functioning is a common feature in many neurological and psychiatric disorders (Casto and Mehta, 2019; Mercadante and Case, 2018). However, due to the complexity of outcome measurements and the challenges in standardizing genetic and environmental factors, the biology of social hierarchies remains largely unexplored, particularly in mice. Therefore, employing appropriate methods to distinguish social hierarchies is crucial for a deeper understanding of their underlying mechanisms.

While no animal models can fully replicate the complexities of natural or human societies, mouse models provide invaluable insights into the mechanisms of social hierarchy and its impact on health and behavior, despite their inherent limitations and challenges. For instance, in wild-type mice, factors such as age, body weight, and baseline stress levels can influence the outcomes of hierarchy tests, but these factors can be controlled through the experimental design. Moreover, research has shown that mice housed with their siblings do not exhibit significant stress responses, suggesting that this social environment can serve as a reliable control condition in studies of social stress (Bartolomucci et al., 2001). The impact of stress during testing, however, remains an important area for further investigation (Larrieu et al., 2017; Park et al., 2018). To minimize the impact of these confounding factors, experiments should ideally match mice for age and body weight and reduce acute stress through habituation and training protocols (Balcombe et al., 2004). Additionally, in genetically modified or experimentally manipulated mice, deficits in social cognition, social memory, or motor abilities may influence test outcomes, necessitating appropriate control experiments before drawing conclusions about social dominance. Because individual behavioral measurements can be influenced by various factors, it is recommended to use more than one method to assess dominance. To this aim, researchers should select multiple measurement methods based on the characteristics of the subjects. For instance, combining the heat source competition test, urine marking, and ultrasonic courtship vocalization measurements can provide a more reliable and valid assessment of social hierarchy by integrating results into a social dominance matrix. Furthermore, the level of environmental enrichment (EE) plays a critical role in shaping social behaviors, including dominance and hierarchy formation. Differences in species, strains, age, sex, type of enrichment, and duration of exposure can all significantly influence EE outcomes, and should be carefully considered when interpreting dominance measures (Kentner et al., 2021).

It is important to note that the winner effect and loser effect may significantly influence the distinction of social hierarchies (Dugatkin, 1997; Oyegbile and Marler, 2005). These effects do not always coexist; in some cases, one effect may occur independently. For example, if animal X defeats animal Z, it may be more likely to defeat animal Y (winner effect), but this does not necessarily mean that animal Z will be more easily defeated in the next interaction (i.e., the loser effect may be absent). Interestingly, related studies have shown that the social hierarchy of male mice is more influenced by previous experiences, making them more susceptible to winner or loser effects (Yan et al., 2024). Recent research in mice highlights the role of dmPFC neurons in mediating the winner effect. Optogenetic manipulation of the dmPFC can induce immediate dominance or submissive behaviors, while its input from the mediodorsal thalamus enables long-term changes in social hierarchy influenced by winning history. This circuit also facilitates the transfer of dominance across contexts, revealing its key role in adaptive social behavior (Zhou et al., 2017). To mitigate the influence of these effects on experimental outcomes, it is advisable to establish fixed intervals between confrontations, ensuring that each individual has sufficient recovery time before and after each encounter. This approach helps to reduce the accumulation of experiential effects between individuals.

In current research, studies on social hierarchies in female mice are relatively scarce, and the impact of sex differences on social hierarchy has not been fully explored. In the wild, reproductive strategies between males and females differ fundamentally (Dean et al., 2006). Males typically exhibit intense intrasexual competition and a strong drive for reproduction, whereas females tend to invest more energy in offspring care, leading to reduced competition for mating opportunities (Bronson, 1979, 1985; Stockley and Bro-Jørgensen, 2011). In mice, the mechanisms by which male mice compete for dominance are well studied, but it remains unclear whether female mice increase aggression to elevate their social rank. However, under laboratory conditions, female mice consistently exhibit low levels of aggression, allowing for the formation of relatively stable social hierarchies (Williamson et al., 2019). Studies indicate that males and females employ different cognitive strategies to maintain social hierarchy stability. Males primarily rely on past experiences to adjust social behavior, whereas females depend more on intrinsic traits such as personality, physique, and sociability. Additionally, testosterone plays a key role in regulating sex-specific social strategies; male mice lacking the Sry gene exhibit female-like behaviors, while transgenic females expressing Sry adopt male-like strategies. Likewise, testosterone deprivation or supplementation can induce male and female mice to adopt opposite social strategies (van den Berg et al., 2015). This experimental design highlights the molecular and hormonal mechanisms underlying sex-specific social strategies, offering valuable insights into the biological basis of sex-related social behavior.

In many polygynous mouse species, identifying the social hierarchy of females is significantly more challenging than that of males. This difficulty arises because females often lack distinct external markers, such as the scars left by male combat. Moreover, female laboratory mice exhibit more subtle competitive behaviors, such as side-pushing and climbing over conspecifics (Clipperton-Allen and Page, 2022; Schuhr, 1987; Williamson et al., 2019), which complicates the identification of social ranks through traditional observational methods. To address these challenges, a range of automated systems has been developed to efficiently identify and analyze the social behaviors of mice. For example, Shemesh et al. (2013) introduced a high spatiotemporal resolution tracking system that monitors the behaviors of four mice in a semi-naturalistic arena containing ramps, nest boxes, and obstacles. By labeling mice with ultraviolet-reactive fluorescent compounds, this system accurately tracked individual behaviors in darkness for several consecutive days, capturing both behavioral details and interaction patterns (Shemesh et al., 2013). Additionally, RFID-based systems have enhanced the capacity to track larger populations of animals, making them particularly useful for long-term studies in complex experimental settings (Freund et al., 2013). These advanced automated systems provide robust tools for studying the social hierarchy of female mice, addressing the limitations of traditional observational methods. However, due to the high cost and complexity of these systems, their application remains limited.

Future research in this field may benefit from further technological advancements and experimental refinements. Current systems primarily focus on the spatial and temporal distribution of behaviors, while future studies could explore the integration of physiological data (e.g., hormone levels) to gain deeper insights into the potential relationships between social hierarchy, individual health, and behavioral adaptation. Additionally, optimizing individual recognition technologies by combining RFID, computer vision, and artificial intelligence algorithms may improve tracking accuracy. If further developed, deep learning techniques might allow researchers to directly analyze the dynamic features of mice, potentially reducing reliance on chemical dyes or implanted devices. As these technologies continue to advance, they may open new avenues for studying social behavior and provide more comprehensive data to support research on female mice social hierarchies.

Although laboratory mice models have made significant progress in uncovering many key mechanisms of animal social hierarchies, it remains unclear how social dominance impacts health. For instance, human SES is a proxy for social hierarchy and is negatively correlated with chronic stress and mortality, but the neural mechanisms underlying this relationship are still under-researched (Marmot et al., 1991). Behavioral interventions aimed at managing psychosocial stress, such as physical activity or social integration, are difficult to standardize across individuals and do not address SES-related factors like income, education, or occupation. On the other hand, pharmacological treatments are often expensive and have adverse side effects. Therefore, as research on the neural and genetic mechanisms underlying dominance behavior advances, clinical and therapeutic approaches to chronic stress may be improved (Wang et al., 2014). Animal models remain a promising option for studying the causal relationships between brain function and psychological stressors related to mental health, such as anxiety, depression, and addiction. Specifically, future research needs to explore how behavioral and pharmacological interventions can modulate neural plasticity to alter brain function (Marmot et al., 1991). For example, if manipulating synaptic plasticity can change a mouse’s social status, would this lead to measurable changes in anxiety, depression, or disease susceptibility?

In summary, mouse models provide a unique platform that allows precise manipulation of environmental variables and in-depth observation of social interactions and competitive behaviors, thereby revealing the neurobiological mechanisms underlying the formation of social hierarchies and offering crucial insights into abnormal social behaviors observed in psychiatric disorders. Furthermore, studying social hierarchies in mice helps to explore the complex relationship between social status and health, such as the close links between social rank, chronic stress, immune function, and neural plasticity. Disentangling the relative roles of these mechanisms is not only crucial for a full understanding of social neurobiology but also provides a scientific basis for creating effective mental health interventions.
